# Transient brain activity dynamics discriminate levels of consciousness during anesthesia

**DOI:** 10.1038/s42003-024-06335-x

**Published:** 2024-06-10

**Authors:** Scott Ensel, Lynn Uhrig, Ayberk Ozkirli, Guylaine Hoffner, Jordy Tasserie, Stanislas Dehaene, Dimitri Van De Ville, Béchir Jarraya, Elvira Pirondini

**Affiliations:** 1 Rehab and Neural Engineering Labs, University of Pittsburgh, Pittsburgh, PA USA; 2 Center for the Neural Basis of Cognition, University of Pittsburgh, Pittsburgh, PA USA; 3 Department of Bioengineering, University of Pittsburgh, Pittsburgh, PA USA; 4 NeuroSpin Center, Institute of BioImaging Commissariat à l’Energie Atomique, Gif/Yvette, France; 5 Cognitive Neuroimaging Unit, INSERM, U992, Gif/Yvette, France; 6 Department of Anesthesiology and Critical Care, Necker Hospital, AP-HP, Université Paris Cité, Paris, France; 7 Neuro-X Institute, Ecole Polytechnique Fédérale de Lausanne, Geneva, Switzerland; 8 Harvard Medical School, Boston, MA USA; 9 Center for Brain Circuit Therapeutics Department of Neurology Brigham & Women’s Hospital, Harvard Medical School, Boston, MA USA; 10 Collège de France, Paris, France; 11 Department of Radiology and Medical Informatics, University of Geneva, Geneva, Switzerland; 12 Université Paris-Saclay (UVSQ), Saclay, France; 13 Neuroscience Pole, Foch Hospital, Suresnes, France; 14 Department of Physical Medicine & Rehabilitation, University of Pittsburgh, Pittsburgh, PA USA; 15 Department of Neurobiology, University of Pittsburgh, Pittsburgh, PA USA; 16 Department of Neurological Surgery, University of Pittsburgh, Pittsburgh, PA USA

**Keywords:** Consciousness, Image processing

## Abstract

The awake mammalian brain is functionally organized in terms of large-scale distributed networks that are constantly interacting. Loss of consciousness might disrupt this temporal organization leaving patients unresponsive. We hypothesize that characterizing brain activity in terms of transient events may provide a signature of consciousness. For this, we analyze temporal dynamics of spatiotemporally overlapping functional networks obtained from fMRI transient activity across different anesthetics and levels of anesthesia. We first show a striking homology in spatial organization of networks between monkeys and humans, indicating cross-species similarities in resting-state fMRI structure. We then track how network organization shifts under different anesthesia conditions in macaque monkeys. While the spatial aspect of the networks is preserved, their temporal dynamics are highly affected by anesthesia. Networks express for longer durations and co-activate in an anesthetic-specific configuration. Additionally, hierarchical brain organization is disrupted with a consciousness-level-signature role of the default mode network. In conclusion, large-scale brain network temporal dynamics capture differences in anesthetic-specific consciousness-level, paving the way towards a clinical translation of these cortical signature.

## Abstract (web summary)

An fMRI study on anesthetized macaque monkeys examines how different depths of anesthesia uniquely affect brain networks temporal dynamics, suggesting that brain dynamics could be used as a cortical signature to gauge consciousness levels clinically.

## Introduction

Recordings of spontaneous brain activity, assessed by resting-state functional magnetic resonance imaging (rs-fMRI), have provided key insights into the rich temporal dynamic and spatial organization of the awake brain^1–6^. Large-scale distributed networks can be extracted from spontaneous fluctuations of the fMRI signals over time. The spatiotemporal organization of these networks can reflect ongoing cognitive efforts and reflect changes in pathological conditions^7^. Recent studies suggested that the temporal dynamics of these networks could inform about the level of consciousness^8–11^.

Detecting residual consciousness in patients remains an open clinical problem for those suffering from disorders of consciousness, vegetative states, or are minimally conscious. Several brain-imaging tests have been proposed to uncover residual signs of consciousness, but the majority of them require subjects to perform difficult and active cognitive tasks^12,13^. Electroencephalography (EEG) has been a thoroughly studied modality to understand consciousness due to its high temporal resolution^14–16^, however its low spatial resolution in particular for subcortical structures limits its clinical capabilities in this field^16–21^. Rs-fMRI could overcome these challenges and large-scale resting-state networks could provide reliable markers of the presence or absence of consciousness. With these premises, rs-fMRI dynamic connectivity has been investigated in sleep^3,19,22,23^, anesthesia in humans^24–27^ and animals^1,4,28,29^, and more recently in unresponsive or minimally conscious patients^8,10,11,30^. Interestingly, all these unconscious states shared hallmark brain changes. Specifically, cortical long-range interactions are disrupted in both space and time and spontaneous network-to-network transitions are less probable as configurations are rigid and tied to the underlying anatomical connectivity. By contrast, wakefulness has been associated with greater global integration and interareal cross-talk and a more flexible repertoire of functional brain configurations departing from anatomical constraints^1,2,8^.

While the dominance of rigid configurations tied to the underlying structural backbone constitutes a putative common signature of unconsciousness, lack of responsiveness can be associated with a variety of brain lesions, varying levels of vigilance, and distinct cognitive levels. We postulate that these differences could be reflected in the spatiotemporal reorganization of specific resting-state networks and/or brain regions. In this regard, previous studies reported varying activations of the visual and of the frontoparietal networks^3^ as well as the posterior cingulate cortex^25,31^ and a reduced activation of the thalamus^24,32^ across different unconscious states. Yet these characterizations remained limited and speculative hampering our capacity to distinguish level of consciousness.

A critical factor hindering the identification of brain network signatures of consciousness is the use of functional connectivity (FC) measures that use spatial and temporal information as mutually dependent. Indeed, the entirety of previous works used a sliding-window technique, where time courses of sets of brain regions are segmented into successive temporal windows and the different assessments of FC are applied to these windows to obtain time-evolving connectivity matrices^1,2^. These time-windowed estimates confine the investigation to slow changes in connectivity^7^. As a result, the dynamic evolution of functional networks interacting over time is not fully captured and their properties remain singular in time.

Here we overcome these limitations by deploying a recently proposed method termed innovation-driven co-activation patterns (iCAPs). iCAPs captures transients (i.e., moments of significant physiological changes in regional activation for each repetition time)^33–35^ through regularized hemodynamic deconvolution of fMRI time series and subsequent clustering of recurrent spatial patterns of transient activity into large-scale brain networks that can be both spatially and temporally overlapping.

Specifically, we adopted the iCAPs framework to recover functional brain networks from fMRI data recorded during different levels of anesthesia-induced unconsciousness in monkeys. For this, we first modified the deconvolution step to account for the hemodynamic response function due to monocrystalline iron oxide nanoparticles (MION). We then validated the translational properties of the identified large-scale brain networks comparing their spatial organization with networks obtained from human rs-fMRI. Finally, we computed temporal properties of these networks and showed that lack of consciousness resulted in longer duration and increased co-occurrence of networks paralleling previously reported reduced brain dynamics. Importantly, the networks co-occurrence and hierarchical organization differed from the conscious wakefulness in an anesthetic-specific configuration with an anesthetic-signature role of the default mode network.

## Results

We scanned 5 rhesus macaques during awake and under anesthesia with three different anesthetics (ketamine, propofol, and sevoflurane) and two different levels of anesthetic depths. Importantly, our sample size is comparable to previous works in NHP with fMRI during anesthesia (see Supplementary Table 1 for a summary of the acquisitions)^36–38^. We used monkey sedation scale and low-density EEG traces to identify the depth of anesthetic for the different anesthetic conditions. In all sessions and animals, the anesthesia level for moderate propofol and moderate sevoflurane corresponded to level 3 on the monkey sedation scale; whereas the anesthesia level for ketamine, deep propofol, and deep sevoflurane corresponded to level 4^2^. We applied total activation (TA), which applies hemodynamically informed deconvolution to the pre-processed fMRI signal of each animal and run separately, to retrieve activity-inducing time courses. Since a MION contrast agent was used during functional scans to increase functional sensitivity^1,2^, we integrated the MION hemodynamic response function (HRF) into the TA pipeline (Supplementary Fig. 1). Then, transients (i.e. moments of activity changes) were computed as the temporal derivative of these activity-inducing signals (i.e., positive or negative spikes) and significant innovation frames were obtained using a two-step spatial and temporal thresholding process. Thresholding was used to identify time points with significantly high/low (i.e. positive/negative spikes) transients. Finally, we used K-means clustering procedure over all animals to label transients and obtain centroids that correspond to large-scale brain networks (i.e., iCAPs) that are potentially spatially overlapping. The optimal number of iCAPs was selected via consensus clustering^3,39^ and the backfitting of the spatial maps of the iCAPs to the activity-inducing time courses revealed iCAPs time series that could be temporally overlapping (Supplementary Fig. 2).

### Typical human large-scale networks were preserved in monkeys

**Fig. 1:**
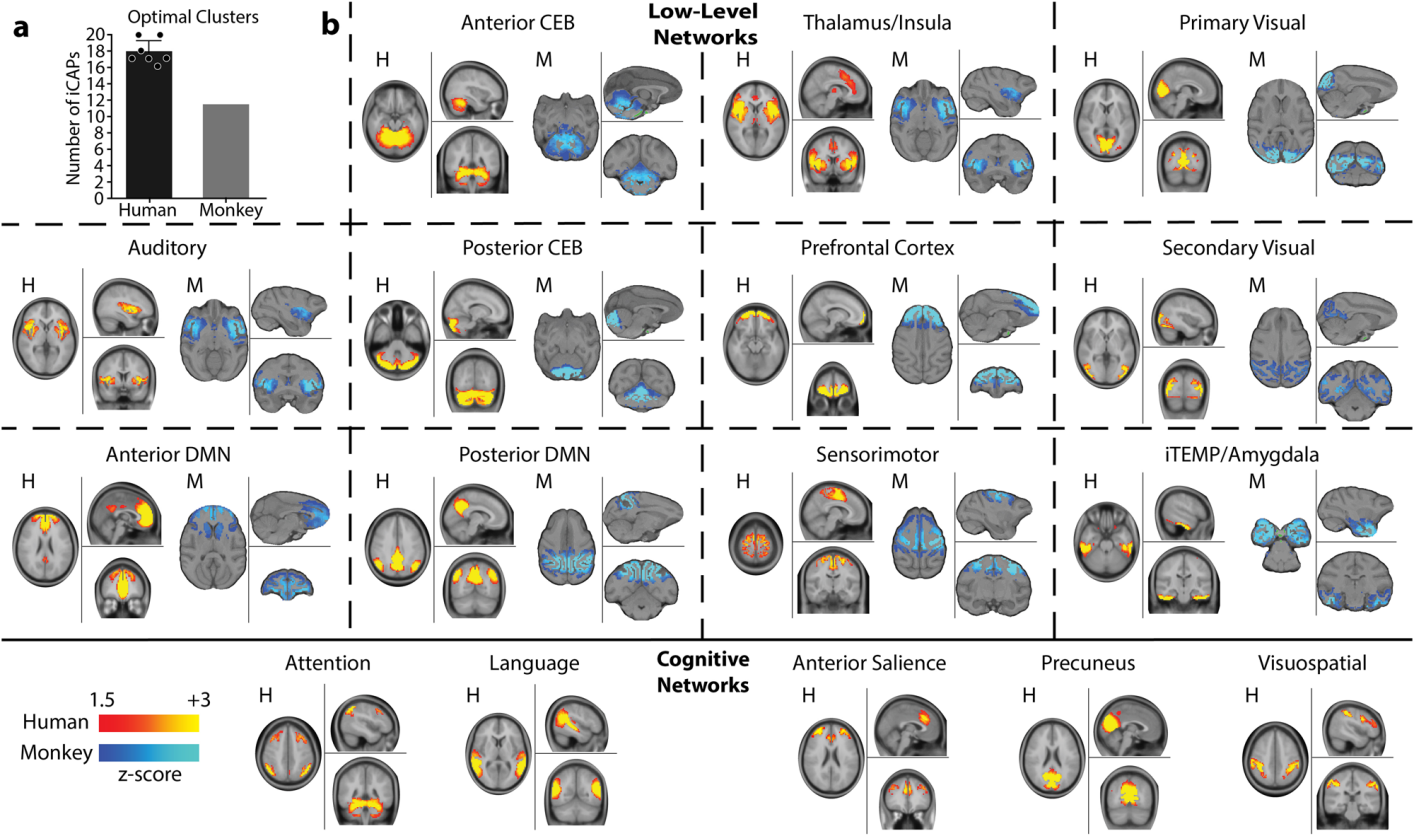
Humans’ and monkeys’ functional networks. **a** Mean number of optimal clusters (iCAPs) found using consensus clustering for 7 studies in humans^3,35,39–43^ (black) and for awake and all anesthesia conditions in monkeys (gray). Error bars represent SD over the 7 studies. **b** *Top rows:* spatial patterns for the most common iCAP spatial clusters aggregated from previous studies in humans (red) compared to iCAP spatial clusters found from the monkeys (blue). All panels show a representative human network, marked with H, on the left, compared to a representative monkey network, marked with M, on the right. Bottom row: high-level cognitive networks that were found only in human studies. For all panels, color bars represent the z-scored voxel values in each spatial iCAP.

In order to validate the translational potential of our results to human subjects, we first visually compared spatial networks extracted using the iCAP framework between humans and monkeys. We considered 7 publications^3,35,39–43^ that computed human iCAPs to gather the predominant human large-scale networks (Supplementary Table 2). We found that generally humans had an optimal number of clusters of *K* = 18 ± 1.5, whereas monkeys had a reduced number of networks (*K* = 11, Fig. 1a). Interestingly, humans and monkeys shared low-level functional networks such as the anterior and posterior cerebellum, primary and secondary visual networks, and auditory network (Fig. 1b). Importantly, also anterior and posterior default mode networks were preserved in monkeys^44,45^. In contrast, monkeys missed higher level cognitive networks such as attention, language, anterior salience and visuospatial networks. The lack of these high-level cognitive networks might explain the difference in optimal number of clusters. Yet, the majority of networks were preserved supporting the translational value of our results and justifying the animal-model choice in our work^46^.

### Slower brain dynamics during unconsciousness

**Fig. 2:**
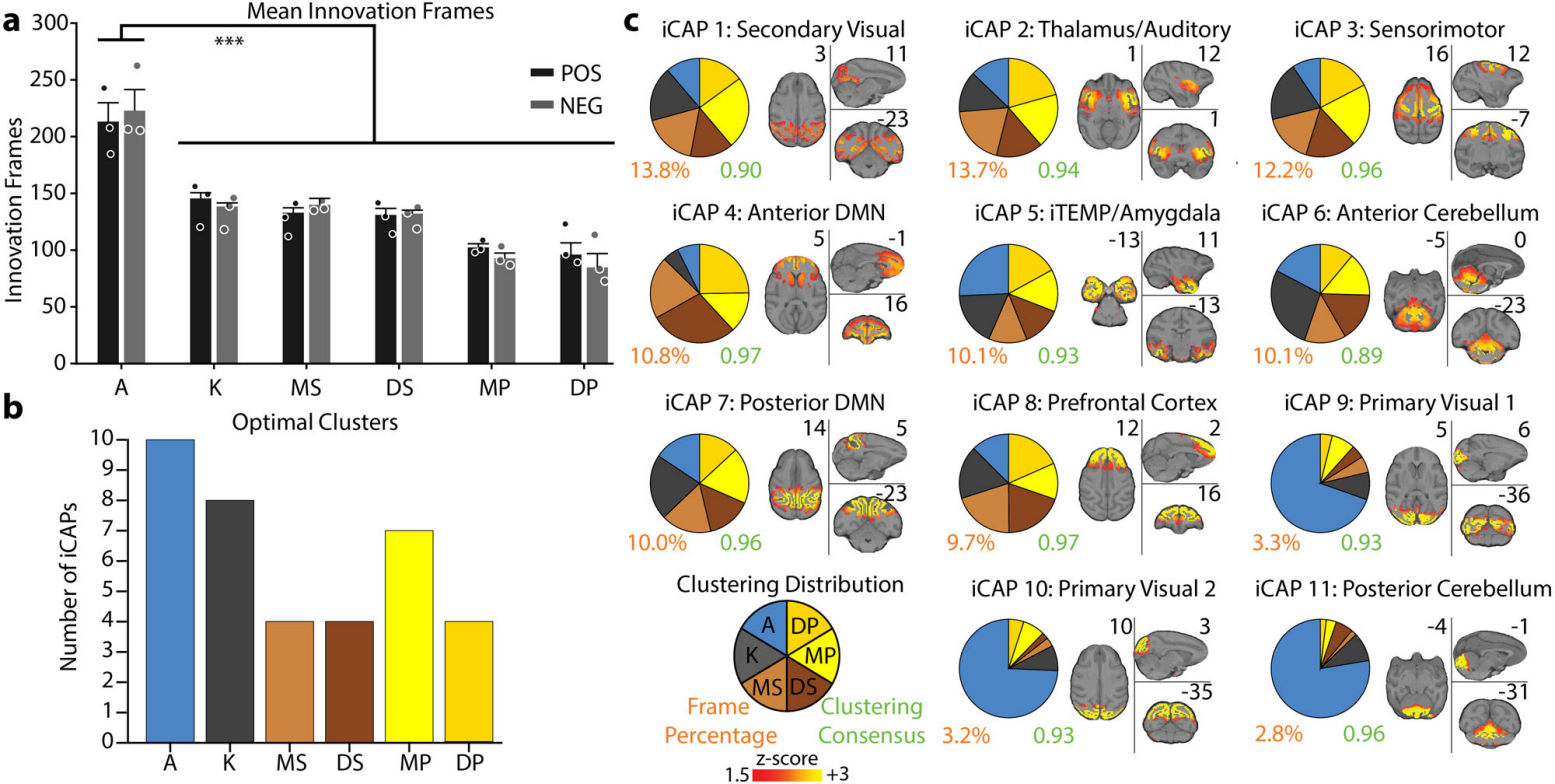
Clustering distributions of significant innovation frames. **a** Bar graph representing the mean number of positive (black) and negative (gray) significant innovation frames for each condition. The error bars represent SEM over each animal and inference on the mean differences is performed by bootstrapping, with *n* = 10,000 bootstrap samples; *** indicates statistical significance of *p* < 0.001 adjusted with Bonferroni correction. (A: awake; K: ketamine anesthesia; MS: moderate sevoflurane anesthesia; DS: deep sevoflurane anesthesia; MP: moderate propofol anesthesia; DP: deep propofol anesthesia). **b** Bar plot representing the optimal number of clusters (iCAPs) found for each individual condition using consensus clustering **(**Supplementary Fig. 3**)**. **c** Spatial patterns for the iCAPs obtained clustering together significant innovation frames from all conditions. The iCAPs are numbered according to the percentage of significant innovation frames that contributed to the recovery of that network (descending order). The percentage of significant innovation frames contributing to each iCAP is shown below the spatial maps in orange font. The cluster consensus of each iCAP is reported in green. In Supplementary Fig. 5, the spatial patterns for the iCAPs extracted clustering the significant innovation frames for each condition separately are shown. For each iCAP, the pie charts indicate the distribution of the significant innovation frames for each condition normalized over the total number of significant innovation frames for each iCAP. MNI coordinates of each brain slice are indicated in black close to each brain. The names of each iCAP are derived according to their correspondence with the CHARM and SARM atlas^91–93^, which are presented in Supplementary Table 3.

We first compared the number of significant innovation frames between awake and anesthetic conditions. We observed a statistically significant difference between the number of significant positive and negative innovation frames in the awake condition as compared to all anesthetics (Fig. 2a, mean ± SEM over animals for awake: 213 ± 16.9, 222 ± 18.8, ketamine: 145 ± 5.0, 138 ± 3.4, moderate propofol: 132 ± 4.3, 139 ± 5.8, deep propofol: 130 ± 6.0, 132 ± 3.0, moderate sevoflurane: 102 ± 3.2, 92 ± 4.9, deep sevoflurane: 96 ± 10.3, 84 ± 12.2 for significant positive and negative innovation frames, respectively). The statistically significant difference in the number of significant innovation frames highlights reduced brain dynamics when the animals were anesthetized.

We then clustered the significant innovation frames to obtain the most prevalent brain spatial patterns (iCAPs) and used consensus clustering to obtain the optimal number of clusters. When clustering significant innovation frames for each anesthetized and awake condition separately, we found that awake had the highest number of clusters (i.e., *K* = 10), whereas the anesthetized conditions ranged from 8 to 4 clusters (Fig. 2b and Supplementary Fig. 3), further supporting the slower brain dynamics during anesthesia.

### Spatial patterns of functional networks were preserved during unconsciousness

When clustering significant innovation frames from all conditions together, we obtained 11 large-scale iCAPs (Supplementary Fig. 4 for the results of the consensus clustering), representing the different functional networks that dominate brain activity across wakefulness and unconsciousness (Fig. 2c and Supplementary Table 3). The iCAPs corresponded to well-known functional large-scale networks obtained both in humans and monkeys with similar and different analysis approaches.

Specifically, the iCAPs included sensory-related networks such as the secondary visual system related to V2 and V3 brain areas, and part of the thalamus (iCAP 1), the primary visual system (iCAPs 9 and 10), the sensory-motor system with a strong activation of the primary motor cortex (iCAP 3), and the auditory network with portions of the thalamus and brainstem (iCAP 2). Importantly, iCAPs 1, 2, and 3 were found to be equally prevalent in all conditions; whereas, iCAPs 9 and 10 were predominantly active during the awake condition. The absence of a network for primary visual areas during unconsciousness parallels previous results on sleep in humans^3^.

**Fig. 3:**
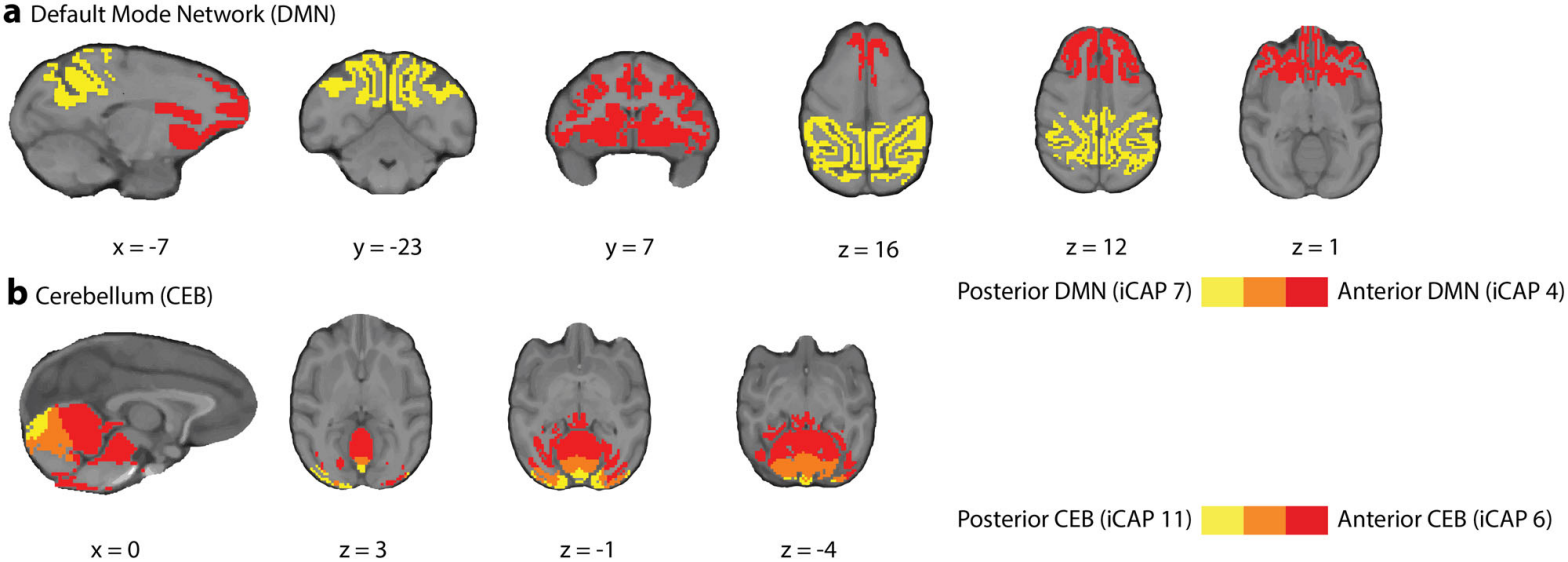
Dissociation of DMN and cerebellum into posterior and anterior parts. **a** Overlay of the iCAPs corresponding to the anterior DMN (red, iCAP 4) and posterior DMN (yellow, iCAP 7) with overlap (orange). **b** Overlay of the iCAPs corresponding to the anterior cerebellum (red, iCAP 6) and posterior cerebellum (yellow, iCAP 7) with overlap (orange). MNI coordinates of each brain slice are indicated in black on the bottom of each brain.

The anterior default mode network (aDMN, iCAP 4) and posterior DMN (pDMN, iCAP 7), instead, showed a similar activation during anesthetized conditions compared to the awake condition. It is of note that the anterior and posterior DMN were separately extracted, which emphasizes a dissociation between the temporal dynamics of these two regions (Fig. 3a). This dissociation of the DMN into subnetworks is commonly observed in other iCAP studies^3^ and in a study of rats under different anesthetic conditions^29^. A similar dissociation was also found for the cerebellum, which split into anterior (iCAP 6) and posterior (iCAP 11) cerebellum (Fig. 3b). Importantly, the anterior portion was found across all conditions, while the posterior cerebellum was present almost exclusively in the awake condition again paralleling previous results on sleep in humans^3^.

Lastly, iCAP 5 and iCAP 8 represented the inferior temporal/amygdala (iTEMP/amygdala) and the prefrontal cortex (PFC) network, respectively, which were composed of significant innovation frames equally distributed across all conditions.

Importantly, spatial patterns of the networks obtained when applying the iCAP framework to each condition separately matched those of the clusters found when conditions were combined as demonstrated by a high cosine similarity (average ± SD over iCAPs and conditions: 0.87 ± 0.13, Supplementary Fig. 5), further supporting that the spatial organization of the networks was preserved during unconsciousness.

### Altered temporal patterns of functional networks revealed consciousness-dependent brain dynamics

We then computed temporal metrics from the iCAPs time courses and considered in particular total and average duration for each iCAP. Importantly, analysis was limited to the first eight iCAPs as the remaining three (iCAPs 9-11) were not present during anesthesia.

**Fig. 4:**
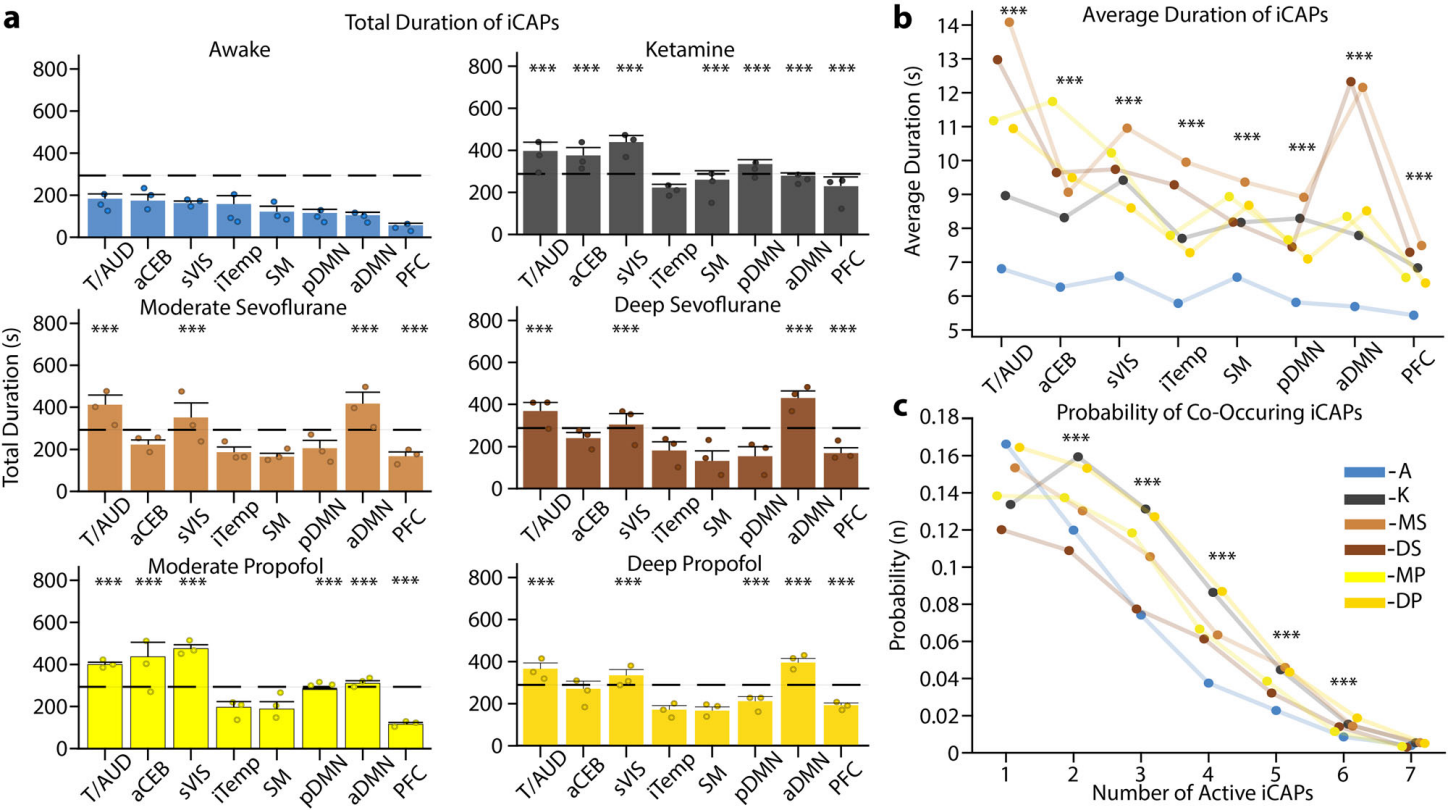
Durations of iCAPs in different conditions and probability of co-occurrences. **a** Total duration of the first 8 iCAPs (in seconds) for each condition in descending order represented in awake. Each condition is represented in its own plot with awake (blue), ketamine (gray), moderate sevoflurane (light brown), deep sevoflurane (dark brown), moderate propofol (light yellow), and deep propofol (dark yellow). Error bars on each bar represent SEM over each animal and the black dashed line represents 25% of total possible duration. Dots represent average values for each animal individually. **b** Average durations (in seconds) of iCAPs reflecting the length of continuous activity**. c** The probability of a different number of iCAPs to overlap is displayed for each condition. For all panels, inference on the mean differences is performed by bootstrapping, with *n* = 10,000 bootstrap samples; *** indicates statistical significance of *p* < 0.001 adjusted with Bonferroni correction.

Interestingly, iCAPs total duration was significantly longer in each of the anesthetized conditions compared to awake (Fig. 4a) in particular for the secondary visual cortex (iCAP 1), thalamus/auditory (iCAP 2), anterior DMN (iCAP 4), and prefrontal cortex (iCAP 8). Yet, iCAPs duration was similar across anesthetics and depths of anesthetic. We then looked at the average duration of iCAPs in a similar fashion (Fig. 4b). Indeed, while total duration only considers the sporadic activation of a network, average duration focuses on the length of continuous activation of each iCAP. Importantly, the continuous activation of iCAPs during anesthesia was also significantly greater than that in the awake condition for all iCAPs. This was consistent with the decrease in the number of significant innovation frames observed in anesthetized conditions, highlighting the direct relation between slower dynamics and increased duration.

To explore beyond the iCAPs’ individual temporal properties, we then evaluated the temporal overlap between iCAPs and computed the probability of having one, two or more iCAPs occurring concurrently (Fig. 4c). iCAPs in the awake condition had a higher probability of occurring alone, whereas during anesthesia they had a higher probability of co-occurring. These results are consistent with iCAPs’ individual temporal properties. Indeed, the longer total and average durations of iCAPs in anesthetized conditions resulted in an increased co-occurrence of iCAPs over time.

In summary, iCAPs temporal properties further supported reduced brain dynamics during anesthesia that was evident in longer duration and increased co-occurrence of brain networks but with no differentiation across anesthetics.

### Networks co-occurrence revealed anesthetic-specific brain dynamics

**Fig. 5:**
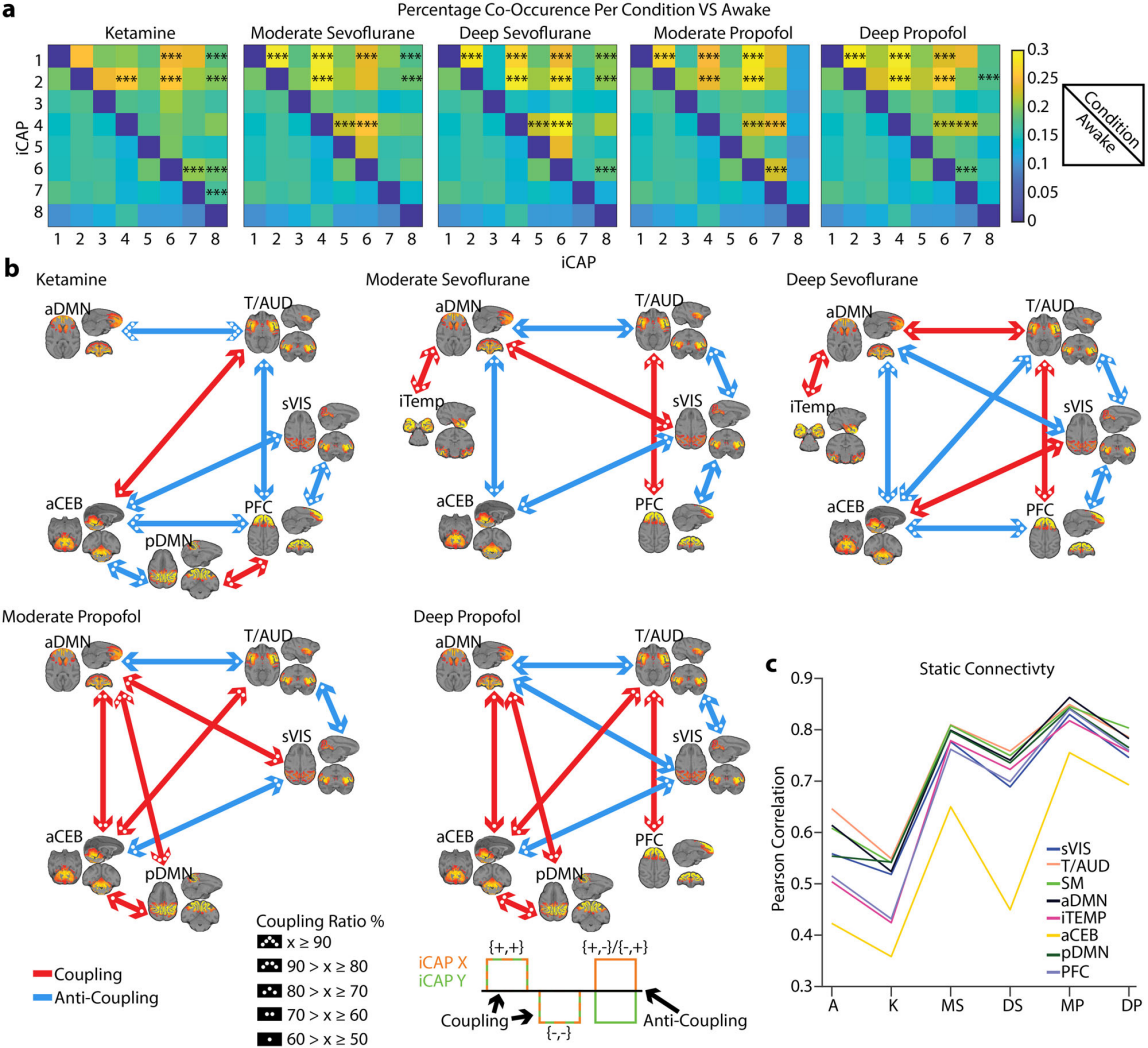
Pairwise coupling between iCAPs across different conditions compared to awake. **a** The co-occurrence pertains to the number of times in a pair of iCAPs both iCAPs were active, divided by the total number of time points where at least one of them was active. Top right of each square is the anesthetic condition and the bottom left of each square is the awake condition. Inference on the mean differences is performed by bootstrapping, with *n* = 10,000 bootstrap samples; *** indicates statistical significance of *p* < 0.001 adjusted with Bonferroni correction. **b** Significant total co-occurrences are broken down into coupling and anti-coupling. Co-occurences dominated by coupling or anti-coupling are indicated in red and blue, respectively, and the ratio between coupling and anti-coupling is indicated by the number of dots in each arrow head. **c** Mean of pairwise Pearson correlation applied to the iCAP time courses. Each color indicates a different iCAP.

To further explore the dynamic interactions across networks, we computed the percentage of co-occurrence for every pair of iCAPs (Fig. 5a), i.e., the number of time-points during which a pair of iCAPs were both active divided by the total number of time-points that at least either one of them was active. Anesthetized conditions had stronger co-occurrence between pairs of iCAPs when compared to the awake condition. Some pairs were shared across anesthetics, whereas others were anesthetic-specific. Indeed, every anesthetic, except ketamine, had a significantly increased co-occurrence for secondary visual cortex (iCAP 1) and thalamus/auditory (iCAP 2) with anterior DMN (iCAP 4) and for secondary visual cortex and thalamus/auditory. Similarly, secondary visual cortex and thalamus/auditory co-occurred with prefrontal cortex (iCAP 8) for ketamine and sevoflurane. Additionally, while the anterior DMN co-occurred with the anterior cerebellum (iCAP 6) for sevoflurane and propofol, the posterior DMN (iCAP 7) co-occurred with the anterior cerebellum for ketamine and propofol. The anterior DMN co-occurred also with the posterior DMN for propofol and with the iTEMP/amygdala (iCAP 5) for sevoflurane. Finally, ketamine had a significantly increased co-occurrence for prefrontal cortex with the posterior DMN and the anterior cerebellum.

We then considered when co-occurrence was significantly different in anesthetized conditions versus awake and looked at the co-occurrence in terms of polarity. Indeed, iCAPs could have a positive or negative activation. We termed the co-occurrence coupling if the two iCAPs were both positively or negatively activated [(+,+),(−,−)] and anti-coupling if the two iCAPs had opposite activation [(+,-),(-,+)] (Fig. 5b). Not surprisingly, some couplings and anti-couplings were common across anesthetics. Specifically, the thalamus/auditory coupled with the prefrontal cortex for all the conditions except moderate propofol and anti-coupled with the anterior DMN, except in deep sevoflurane (coupling). The secondary visual cortex, instead, is anti-coupled with the thalamus/auditory except for ketamine. Interestingly, the coupling between the secondary visual cortex and the anterior DMN was positive for light depths of anesthetic (moderate propofol and moderate sevoflurane) and negative for deeper depths of anesthetic (deep propofol and sevoflurane). Under ketamine, moderate, and deep sevoflurane the prefrontal cortex was anti-coupled with the secondary visual cortex and the anterior cerebellum (not for moderate sevoflurane) showing similar interactions across these anesthetics in particular during deep anesthesia. However, ketamine was distinct from the other anesthetics as it had a higher proportion of anti-coupled pairs representing a strong difference in brain dynamics. Finally, the anterior DMN and anterior cerebellum were positively coupled under propofol and negatively under sevoflurane.

Importantly, couplings and anti-couplings between iCAP pairs paralleled findings obtained with a classic functional connectivity (FC) analysis. Indeed, we computed Pearson correlation of the iCAPs time courses and we found that pairwise FC across iCAPs increased in all the anesthetics as compared to awake except for ketamine (Fig. 5c). Ketamine’s reduced correlation between networks matched its stronger anti-coupling of iCAPs. Interestingly, the anterior cerebellum was unique as compared to other iCAPs showing a reduced connectivity, which parallels its susceptibility to be anti-coupled with other networks.

In summary, while generally iCAPs co-occurred more during unconsciousness for all anesthetics, different drugs and more importantly different levels of anesthetic-induced unconsciousness altered coupling and anti-coupling between specific iCAPs, supporting the need to assess spatiotemporal networks interaction to discern alertness levels.

### Network hierarchical organization revealed an anesthetic-specific role of the DMN

**Fig. 6:**
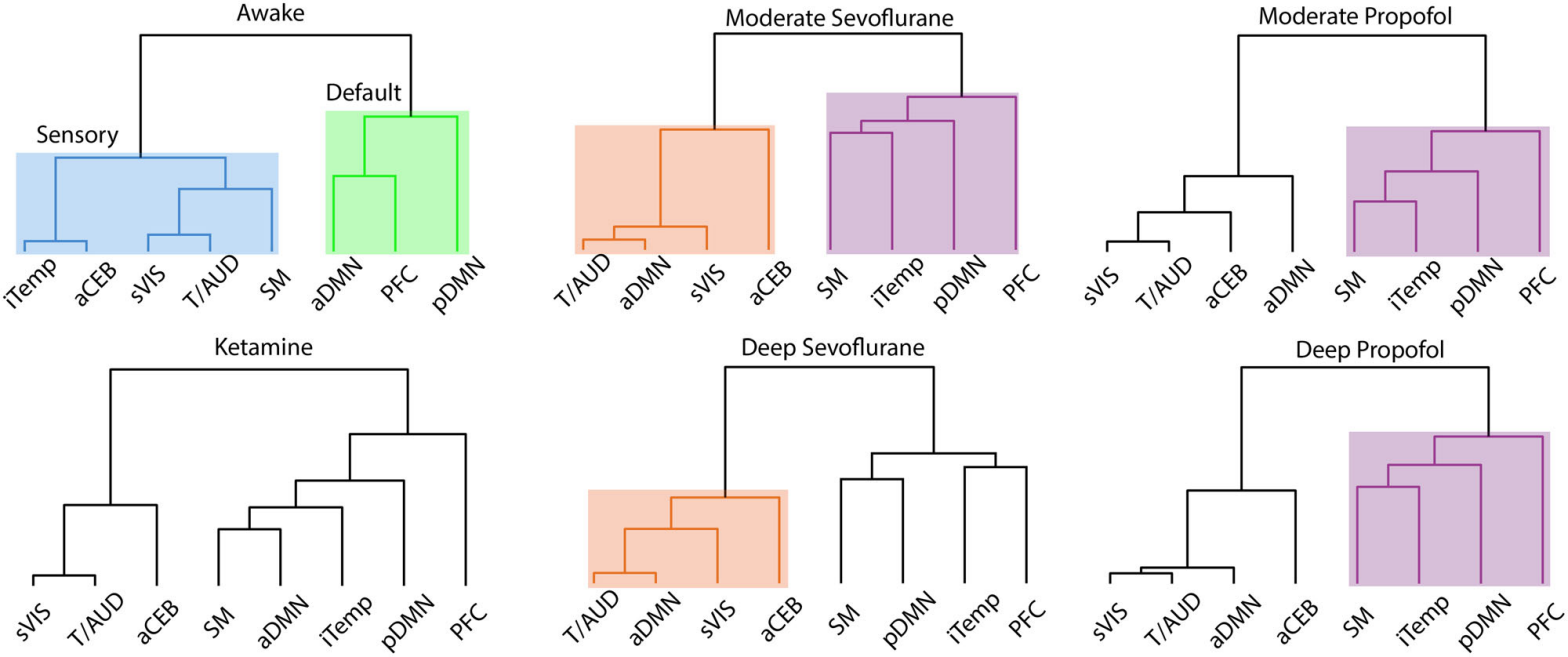
Hierarchical clustering of iCAPs according to their temporal overlap. The dendrogram minimizes the distance at each leaf with respect to the neighboring leaf, clustering the most similar iCAPs. Each condition is represented by their own tree with some branches highlighted by unique colors to emphasize similarities or differences for each cluster. Awake is the only condition separated into a sensory and default networks; whereas the other conditions have unique formations.

Co-occurrence is evaluating when only two iCAPs occur at the same time, but within the iCAP method more than two iCAPs can occur at each timepoint. Therefore, we expanded our analysis to all possible combinations of iCAPs and captured the most probable occurrences using hierarchical clustering. This analysis ensures that we explore whole brain dynamics instead of limiting our view to pairs of networks. The dendrogram reflects a hierarchy of iCAPs based on their frequency of occurrence together (Fig. 6). In awake, similarly to previous results in humans^35^, iCAPs were grouped into two large clusters of sensory and default-mode networks. Interestingly, this division was not preserved during anesthesia. Indeed, except for ketamine, the anterior and posterior DMN split into two different clusters further supporting the strong dynamical dissociation of the DMN into subnetworks. In the anesthesia conditions, except for ketamine, the anterior DMN was clustered with the secondary visual, thalamus/auditory, and anterior cerebellum. Instead, in the other large branch, there was a consistent organization of the posterior DMN, prefrontal cortex, iTEMP/amygdala, and sensory-motor cortex. Within this split light and deep propofol and moderate sevoflurane shared the same hierarchical organization as opposite to deep sevoflurane. Ketamine, instead, was unique as it shared the same organization of this split but included the anterior DMN.

In summary, unconsciousness disrupts the hierarchical organization of brain dynamics dissociating the anterior and posterior DMN. Importantly, the association of the latter with other networks appears to be pivotal in differentiating anesthetics and level of sedation.

## Discussion

Recent experiments in sedated humans, rats, and monkeys^24,25,29,47^ have shown that under general anesthesia spontaneous brain activity converges to a few or even a single dominant brain dynamical pattern. This reduced dynamic was confirmed in unresponsive or minimally conscious patients^8,10,11,30^ validating the use of pharmacologically induced anesthesia models to study loss of consciousness. However, these decreased dynamics may not simply derive from an overall reduction of functional connectivity, but it could be the result of a complex orchestration of preserved and suppressed functional networks^48^. This composition may be occulted when using static FC or sliding window methods^1,2^, challenging our ability to distinguish levels of consciousness. Indeed, the correlation between two distinct brain areas exhibiting both positive and negative correlations in distinct time-windows would result in a low FC, despite the dynamic synchronization existing between these regions. Here, we overcome the limitations imposed by static FC or sliding windows methods by clustering moments of significantly changing brain activity from multiple fMRI sessions while animals at rest were awake or under anesthesia to extract large scale brain networks or iCAPs. The use of transient fMRI activity allowed us to obtain temporally overlapping spatial networks and to compute their time courses at the resolution of repetition time. We established that functional brain networks in the unconscious states preserved their spatial organization compared to the awake state; yet they had fewer dynamic fluctuations than conscious states, which resulted in longer temporal activation and higher co-activations. Importantly, network co-occurrence showed anesthetic-specific trends and a hierarchical organization specific to the depth of anesthetic. Altogether, these results advance the use of rs-fMRI to detect the presence or absence of consciousness. Here, we discuss our findings with an emphasis on key anatomical brain structures and temporal measures that distinguish different anesthetics and sedation depth.

During unconsciousness, the amount of transient brain activity significantly decreased as compared to awake resulting in a reduced number of clusters, which was explained by the absence of activation of primary visual cortex and posterior cerebellum. The lack of a primary visual cortex network might be explained by the difference in eyes-opened during awake, and eyes closed when under anesthesia. The absence of the posterior, but not the anterior cerebellum, instead, might be explained by the organization of the cerebellum lobules. Indeed, the anterior lobules have been reported to be responsible for the sensorimotor domain, while the posterior ones for the cognitive domain^49^. Additionally, previous works reported a lack of the posterior cerebellum network during sleep^3^ and a marked correlation of larger lobules (i.e., anterior region of the cerebellum)^50^ with slow-wave sleep and slow spindles in different sleep stages^51^. Yet the anterior cerebellum showed a reduced correlation of activity compared to all the other functional networks across all anesthetic conditions highlighting a unique functional organization of the anterior lobules. Importantly, the spatial organization of the other brain networks was preserved.

To explain how the predominance of temporal reorganization can explain loss of consciousness during anesthesia while anatomical networks are preserved, we showed a global increase in simultaneous coupling and anti-coupling of the different brain networks. These results are in line with previous studies^52^ and commonly interpreted to reflect reduced consciousness during sleep^53^. We here showed that a similar mechanism might occur during anesthesia further supporting the idea that consciousness is not the persistence of functional brain networks, but rather the degree of interactions among them^29,52,54–56^. This lack of integrity and synchronization could be due to a reduced activation of the thalamus. Yet, the role of the thalamus in inducing loss of consciousness remains controversial. Indeed, several studies show that anesthesia-induced loss of consciousness is mostly associated with a change in cortical correlation rather than with an alteration of thalamic activity^57,58^. On the other hand, because of its pivotal role in the exchange of information between the periphery and cortex, the thalamus has been hypothesized to be essential in inducing loss of consciousness^32^. In this regard, a previous work in rats under multiple conditions of anesthesia found consistent suppression of the thalamo-cortical pathways suggesting less dynamic functional connectivity^29^. Additionally, recent studies showed that stimulation of the thalamus during anesthesia restored arousal and wake-like neural processing as depicted by brain fluctuations similar to the awake state^9,59,60^. The spatiotemporal organization of iCAPs further supports this second hypothesis. Indeed, both iCAPs 1 and 2, which included portions of the thalamus, presented the strongest temporal changes during loss of consciousness. Specifically, they fluctuated with the longest average durations and had an increased co-occurrence with other networks, particularly with the anterior DMN and the prefrontal cortex. These areas are highly coupled together through the lateral forebrain bundle, which connects the temporo-parietal DMN nodes, whose connectivity is believed to be essential for conscious awareness^61,62^. Interestingly, these cortical areas also had a significantly longer total duration during anesthesia further suggesting that loss of consciousness might induced by a reduced activation of the thalamus that caused a slowing down of the prefrontal cortex and DMN.

Importantly, while the reduced fluctuations in the thalamo-prefrontal pathways was a common signature of anesthesia-induced loss of consciousness no matter the initial molecular mechanism of the anesthetic, co-activations of other brain areas were, instead, anesthetic-specific. In particular, the activation of the anterior cerebellum differentiated the most across opiates. While the cerebellum has long been considered a marginal target for general anesthesia^63^, recent studies suggested a possible involvement of this structure explained by its strong connectivity with prefrontal and frontal cortical areas^64^. Our results further support this hypothesis. Indeed, the anterior cerebellum anti-coupled with the anterior DMN when unconsciousness was induced with sevoflurane, but these networks positively coupled during propofol-induced anesthesia. The anterior cerebellum likewise anti-coupled more with the prefrontal cortex under ketamine and sevoflurane sedation than during the awake condition. Importantly, these more probable cortical co-activations of the anterior cerebellum during anesthesia as compared to the awake condition reflect a reduction of low-frequency fluctuations in the frontal regions and cerebellum, which is consistent with frontal to sensory-motor cortical disconnection as a possible mechanism of loss of consciousness^65^.

Also, the hierarchical organization of the networks was unique between conscious and unconscious states and differentiated levels of alertness. Indeed, brain activity during consciousness arranged into components related to sensory and attention, matching organization shown in previous studies of awake humans^3^. During unconsciousness, instead, this organization was altered in particular at the level of the DMN connectivity. Specifically, the anterior and posterior parts of the DMN split into two different hierarchical clusters: the anterior DMN reorganized with the secondary visual, thalamus/auditory, and anterior cerebellum; while the posterior DMN clustered with the prefrontal cortex, iTEMP/amygdala and sensory-motor cortex. These findings parallel previous works showing that functional correlation decreased in the DMN during all the anesthetic conditions, as compared to the awake condition^66–68^. Similarly, the DMN dissociates into posterior and anterior parts upon reaching deep sleep^56,69^ with an increased activation of the posterior DMN^3^. These similarities suggest a common neurophysiological role of the posterior DMN in inducing unconsciousness between sleep and anesthesia. Importantly, within the hierarchical split containing the posterior DMN, moderate and deep propofol, and moderate sevoflurane shared the same hierarchical organization as opposite to deep sevoflurane. Finally, ketamine had a unique hierarchy of iCAPs with the anterior and posterior DMN in the same split together with the prefrontal cortex, iTEMP/amygdala and sensory-motor cortex. If we assume a finer definition of level of alertness with moderate propofol the lowest depth of anesthesia and deep sevoflurane and ketamine the strongest, our results hint to a critical role of the posterior DMN; i.e., the posterior cingulate cortex, in determining the level of consciousness.

Overall these results have important clinical implications. First of all, it is important to highlight here the rich literature that used EEG to determine level of consciousness^15,16,57,70–72^. While EEG is critical during surgical procedures to monitor the depth of the anesthesia and the level of consciousness and for longitudinal assessments over time, EEG cannot directly access the thalamo-cortical and cerebellar-cortical functional connectivity that seems to be critical in maintaining consciousness^16–21^. Clinical application, therefore, needs to be complemented with high spatial resolution fMRI exploration. In this regard, previous fMRI works in humans using the iCAP method reported large-scale brain networks that are consistent with our results demonstrating similar cognitive processes and neurobiological correlates in humans and non-human primates which enables translation of discoveries. Common FC network structures across species were reported also in other studies further supporting the generalizability of our work to patients^4,47,73–77^. Importantly, the similarity across networks was preserved even if our approach uses a custom MION HRF instead of the standard HRF. Additionally, our results nicely parallel finding from a previous work that applied iCAPs to fMRI data acquired in humans during different levels of sleep^3^ highlighting not only the shared neurophysiological mechanisms between sleep and anesthesia^3,22,23,50,51,53,56,69^, but more importantly the translational potential of our results to human subjects. All together these similarities open up the possibility that residual consciousness could be monitored through the dynamics of brain activity and tentatively suggests biomarkers of conscious activity. Our findings could help ameliorate the accurate diagnosis of patients with disorders of consciousness.

In conclusion, dynamic functional connectivity for fMRI has been investigated in different sleep stages^3,22,23^, under different anesthetic sedation, and more recently in unconscious patients^8,10,11,30^ showing a decreased brain dynamics. However, the investigation regarding the affected brain areas remained limited and speculative, thus hindering our capacity to distinguish alertness. Here we deployed the iCAPs framework that has the unique ability to extract spatially and temporally overlapping functional networks. We observed a clear dissociation of both the DMN and cerebellum into anterior and posterior networks. Thanks to these features, we identified key brain areas responsible for loss of consciousness and with different effects depending on the anesthetic and the level of consciousness: the default mode network, the anterior cerebellum, and the thalamus. Specifically, the activation of the thalamus may play a central role, common to all anesthetics, on reducing the incoming information flow from the periphery that reduces the whole-brain dynamics. Simultaneously, a decreased activation of the anterior cerebellum could cause a disconnection with the frontal regions, with an anesthetic-specific differentiation between its connections to the anterior DMN and the prefrontal cortex. Finally, the posterior DMN may be key in regulating the desynchronization of multiple networks during different levels of consciousness.

## Materials and methods

### Animals

5 rhesus macaques (Macaca mulatta), 1 male (monkey J), and 4 females (monkeys A, K, Ki, and R), 5–8 kg, 8–12 year of age, were tested; three for each arousal condition (awake: monkeys A, K, and J; propofol anesthesia: monkeys K, R, and J; ketamine anesthesia: monkeys K, R, and Ki; sevoflurane anesthesia: monkeys Ki, R, and J). See Supplementary Table 1 for details on the exact number of acquisitions per animal and condition. All procedures were conducted in accordance with the European Convention for the Protection of Vertebrate Animals used for Experimental and Other Scientific Purposes (Directive 2010/63/EU) and the National Institutes of Health’s Guide for the Care and Use of Laboratory Animals. Animal studies were approved by the institutional Ethical Committee (Commissariat à l'Énergie atomique et aux Énergies alternatives; Fontenay aux Roses, France; protocols 10-003 and 12-086). Part of the data used in this work were already presented in refs. ^1,2^. Anesthesia protocols were the same of these previous works. We have complied with all relevant ethical regulations for animal use.

### Anesthesia protocol

The monkeys were administered anesthesia using either ketamine^78^, propofol^1,78^, or sevoflurane. We gauged the depth of anesthesia by employing the monkey sedation scale, which considers spontaneous movements and responses to various external stimuli such as shaking, prodding, toe pinching, and assessing the corneal reflex. Clinical scores were determined at the outset and conclusion of each scanning session, coupled with continuous electroencephalography monitoring.

For ketamine anesthesia, the monkeys received an initial intramuscular injection of ketamine (20 mg/kg; Virbac, France) for induction, followed by a continuous intravenous ketamine infusion (15–16 mg kg^–1^  h^–1^) to maintain anesthesia. Atropine (0.02 mg/kg intramuscularly; Aguettant, France) was administered 10 minutes prior to induction to reduce salivary and bronchial secretions. For propofol anesthesia, the monkeys were scanned at moderate propofol sedation and deep propofol anesthesia levels. For this, the animals were trained to receive an intravenous propofol bolus (5–7.5 mg/kg; Fresenius Kabi, France) for anesthesia induction, followed by target-controlled propofol infusion (moderate propofol sedation: 3.7–4.0 µg/ml; deep propofol anesthesia: 5.6–7.2 μg/ml) based on the “Paedfusor” pharmacokinetic model^79^. Despite the “Paedfusor” model being validated in humans, we previously applied it to macaque monkeys, observing stable clinical scores and electroencephalography activity during propofol anesthesia sessions^1,78^. Finally, for sevoflurane anesthesia, the monkeys were subjected to moderate and deep sevoflurane anesthesia. The induction of anesthesia was done by intramuscular ketamine injection (20 mg/kg; Virbac), followed by sevoflurane administration (moderate sevoflurane anesthesia: sevoflurane inspiratory/expiratory, 2.2/2.1 volume percent; deep sevoflurane anesthesia: sevoflurane inspiratory/expiratory, 4.4/4.0 volume percent; Abbott, France). We waited a minimum of 80 minutes^80^ to elapse before initiating scanning sessions during sevoflurane anesthesia to ensure the elimination of the initial ketamine injection. To prevent movement-related artifacts during magnetic resonance imaging acquisition, a muscle-blocking agent (Cisatracurium, 0.15 mg/kg bolus intravenously, followed by continuous intravenous infusion at a rate of 0.18 mg kg^–1^ h^–1^; GlaxoSmithKline, France) was co-administered during the ketamine and deep propofol sedation sessions. Importantly, the muscle-blocking activation has no impact on the hemodynamic response as it does not affect the vasculature^81^. Indeed, Cisatracurium acts exclusively on the level of the neuro-muscular junction, relaxing only skeletal muscles with no vascular effects nor neuronal effects.

In all anesthesia experiments, the monkeys were intubated and ventilated. Vital signs such as heart rate, noninvasive blood pressure (systolic/diastolic/mean), oxygen saturation, respiratory rate, end-tidal carbon dioxide, and cutaneous temperature were continuously monitored (Maglife, Schiller, France) and recorded online (Schiller).

### Electroencephalography

To assess the depth of anesthesia, we utilized low-density scalp EEG with a MRI compatible system and custom-made caps, as detailed previously^1^. Importantly, no EEG acquisitions were possible during the awake condition because of the difficulty to use the system with awake large animal. Our analysis was conducted in real-time through visual inspection of EEG patterns following established guidelines^78^. For ketamine, sedation level was 4, which was characterized by intermittent polymorphic δ activity (0.5–2 Hz) of large amplitude, overlaid by low-amplitude β activity^70^, and an increase in γ power (30–100 Hz)^82^. For propofol, sedation levels were 3 (moderate propofol sedation) featuring diffuse and wide α waves, along with anterior theta waves^83^, and level 4 (deep propofol) marked by diffuse delta waves, low-amplitude waves^71^, and anterior α waves (10 Hz)^84^. For sevoflurane, sedation levels were 3 (moderate sevoflurane) featured by elevated frontal delta, α, and β waves^72^, and level 4 (deep sevoflurane anesthesia) characterized by diffuse delta waves and anterior α waves^72^.

### Functional magnetic resonance imaging data acquisition

The data were collected between July 2011 and August 2016. Monkeys were scanned on a 3-Tesla horizontal scanner (Siemens Tim Trio, Germany) with a single transmit-receiver surface customized coil. Each functional scan consisted of gradient-echoplanar whole-brain images (repetition time = 2400 ms; echo time = 20 ms; 1.5-mm^3^ voxel size; 500 brain volumes per run). Before each scanning session, monocrystalline iron oxide nanoparticle (MION, Feraheme, AMAG Pharmaceuticals, USA; 10 mg/kg, intravenous) was injected into the monkey’s saphenous vein^1^. For the awake condition, monkeys were implanted with a magnetic resonance compatible headpost and trained to sit in the sphinx position in a primate chair without performing any task^1,85^. The eye position was monitored at 120 Hz (Iscan Inc., USA) only for the awake condition. For the anesthesia sessions, animals were positioned in a sphinx position, mechanically ventilated, and their physiologic parameters were monitored.

### Functional magnetic resonance imaging preprocessing

Functional images were preprocessed using the Pypreclin pipeline for monkey fMRI^86^. Images were slice-time corrected with FSL slice timer function (FMRIB’s Software Library – FSL, Oxford, U.K)^87^. B0 inhomogeneities and B1 field were corrected using the SyN function and N4 normalization of the Advanced Normalization Tool (ANTS). Images were reoriented, realigned, and rigidly co-registered to the anatomical template of the macaque Montreal Neurologic Institute (MNI, Montreal, Canada) space with use of JIP align (http://www.nmr.mgh.harvard.edu/~jbm/jip/, Joe Mandeville, Massachusetts General Hospital, Harvard University, MA, USA) and Oxford Centre Functional Magnetic Resonance Imaging of the Brain Software Library software (United Kingdom, http://www.fmrib.ox.ac.uk/fsl/; accessed 4 February 2018)^85^. The data were denoised using the non-human primate adapted ICD-FIX command (https://github.com/Washington-University/NHPPipelines) for spatial Independent Component Analysis (ICA, i.e. melodic) followed by automatic classification of components into ‘signal’ and ‘noise’. We then applied spatial smoothing using an isotropic Gaussian kernel of 3 mm full width at half maximum.

### Total activation and the iCAPs framework

In order to extract large-scale brain networks and their temporal characteristics we deployed the iCAP pipeline^33,35,40,42,43,88^ (Supplementary Fig. 2). We first implemented the Total Activation framework which takes the pre-processed fMRI time series of each voxel and applies MION informed deconvolution (Supplementary Fig. 1) to retrieve an activity-inducing signal that shows block-type activation patterns (without any prior knowledge of its timings, Supplementary Fig. 2). More details about the implementation the TA framework can be found in Karahanoğlu et van De Ville, 2015^35^.

Then, for each activity-inducing time courses we removed the first ten volumes (i.e., 490 volume kept in total per run) and significant activation change-points (i.e., transients) were computed as the temporal derivative of these activity-inducing signals. Specifically, the derivative of the signal results in negative or positive spikes that represents the positive and negative transient frames. Significant innovation frames (i.e. frames with significant transitioning activities) were selected with a two-step thresholding procedure with temporal and spatial thresholds selected based on previous works^33–35,40,42,43^. This two-step thresholding allowed to select only frames that contained significantly transient activity and to avoid including spurious connectivity patterns. The purpose of the temporal thresholding was, for each voxel, to find the time points where the activity was significantly high/low (i.e. positive/ negative). To determine the temporal threshold a surrogate data set, created by phase-randomization of the real data, was used to build a surrogate distribution where the lowest 1^st^ and highest 99^th^ percentile was used to select significant voxels. We did that for all voxels, and obtained, for each time point, a map of significant positive and negative transients (regions that are jointly activation, positive, and regions that jointly deactivation, negative). We then applied spatial thresholding on these maps, and only select those that have more than 5% of significant voxels (i.e., significant innovation frames).

Significant innovation frames across all animals, sessions, and anesthetics were then fed into a temporal k-means clustering to obtain large-scale resting state networks, the iCAPs. We also clustered significant innovation frames for anesthetic conditions separately. The iCAPs found when clustering each condition individually were compared with the iCAPs found when conditions were clustered all together using cosine similarity of their spatial maps. In both cases, the optimal number of clusters was determined by consensus clustering^89^. The method involved subsampling of the data and multiple runs of the clustering algorithm. The number of clusters was evaluated from *K* = 5–15. We chose *K* = 11 to be the optimal number of clusters by considering the agreement between three criteria. First of all, we monitored the consistency of each frame being grouped into the same cluster over every subsample to create a consensus matrix. A perfect consensus would result in a block diagonal with each block representing a cluster. Secondly, we looked at the slope of the cumulative distribution function (CDF) of the consensus clustering matrices. In the case of perfect consensus, the CDF curve would look like a step function as the consensus matrix would only contain 1 and 0. Instead, the higher the slope of the CDF the less stable the clusters are. We lastly looked at the area under the curve (AUC) of the CDF to find if the CDF increases or decreases as a function of K. When going from a perfect consensus for one *K* to a perfect consensus of a larger K, the AUC would show a large increase. However, going from a perfect consensus to a less good consensus would lead to a smaller increase in the AUC. The idea is to then look at the AUC plot as a function of K and choose K such that the AUC is large and decreases drastically after.

Finally, run-specific iCAP time courses were obtained by transient-informed spatiotemporal back-projection of the 11 spatial maps (i.e. the large-scale rs-fMRI networks extracted when clustering together significant innovation frames concatenated across all sessions) onto the individual activity-inducing signals. For each animal and run the iCAPs time courses were Z-scored in order to obtain positive and negative activation signs. iCAP positive/negative activations reflect the time-points when an iCAP is at least 1.5 standard deviations above/below the overall mean amplitude of iCAPs. The choice of this particular threshold was motivated by previous works that implemented TA and iCAP framework^3,40^. For each iCAP and session, we then computed the total duration of each iCAP occurrence as the number of time points that an iCAP was active or de-active. We also computed the average duration measured in seconds: the length of time that an iCAP is continuously active. We then evaluated the pair-wise iCAP percentage of co-occurrences which is represented by the number of time-points during which a pair of iCAPs were both active divided by the total number of time-points that at least either one of them was active. We also consider the signs of the co-occurrence. Specifically, we considered the pair of iCAPs to be coupled (coupling) if both iCAPs had positive or negative activation or anti-coupled (anti-coupling) if one iCAP was positively/negatively activated and the other had opposite activation (i.e., negatively/positively, respectively). Lastly, to measure the temporal overlap between groups of iCAPs, we counted different combinations of iCAPs occurring at each time instance. We then applied hierarchical clustering of iCAPs using the observed combinations as features. Matlab (Mathworks, USA) code for the application of the whole framework can be found at https://www.c4science.ch/source/iCAPs.

### Statistics and reproducibility

Testing for statistical differences of any iCAP measure was done through a bootstrapping method^90^. A nonparametric approach that makes no distributional assumptions on the observed data. Bootstrapping instead uses resampling to construct empirical confidence intervals (Cis) for a quantity of interest. For each comparison we constructed bootstrap samples by drawing with replacement from the observed measurement. We calculated the null distribution of the observed difference by creating 10,000 bootstrap samples. A 95% CI for the observed difference was obtained by identifying the 2.5th and 97.5th quantiles of the resulting null distribution. The null hypothesis was rejected if 0 was not included in the 95% CI. If more than one comparison was being performed then Bonferroni correction was used. For the analysis of temporal characteristics, we only included the first eight iCAPs as they were equally prevalent across all conditions.

### Supplementary information

Supplementary Information

Description of Additional Supplementary Materials

Supplementary Data

## Data Availability

The source data underlying the graphs in the manuscript are shown in Supplementary Data. Raw data are uploaded in the BIOPROJ – Nextcloud repository. Access to the data will be provided from the corresponding author on reasonable request. Data supporting the findings of this manuscript are available from the corresponding authors upon reasonable request.

## Code availability

fMRI data were analyzed using a publicly available algorithm. https://miplab.epfl.ch/index.php/software/total-activation.
