# Supplementary material for: Transient brain activity dynamics discriminate levels of consciousness during anesthesia
Source: Commun Biol. 2024 Jun 10;7:716. doi: 10.1038/s42003-024-06335-x (PMC11164921; doi:10.1038/s42003-024-06335-x)
Supplement: Supplementary file 1 — Supplementary Information [file 42003_2024_6335_MOESM1_ESM.pdf]

Supplementary Figures and Tables

	Awake	Ketamine	Moderate Sevoflurane	Deep Sevoflurane	Moderate Propofol	Deep Propofol	Totals
MK-J	18	0	5	2	1	6	32
MK-Ki	0	7	10	8	0	0	25
MK-A	4	0	0	0	0	0	4
MK-K	9	5	0	0	7	9	30
MK-R	0	10	8	10	12	12	52
Totals	31	22	23	20	20	27	143

Supplementary Table 1: Number of sessions for each monkey and for awake and each type of anesthetic.

**Paper**	Karahanoglu 2015	Zoeller 2018	Zoeller 2019	Zoeller 2021	Piguet 2021	Tarun 2020	Pirondini 2022
**TR**	1.1	2.4	2.4	2.4	2.1	2.1	2
**Number of iCAPs**	20	18	17	17	20	17	16
**Auditory**							
**Language**							
**Executive**	anterior					left /right	
**Attention/ FPN**		left/ right					
**Primary Visual**			2	2	3		3
**Secondary Visual/Higher Visual**							
**DMN**							
**aDMN/ACC**							
**pDMN**							
**Salience**	anterior		anterior	anterior	anterior/ full (2)		anterior
**OFC**							
**Precuneus/**							

**PCC/Thalamus**							
**CEB**							
**aCEB**							
**pCEB**							
**Visuospatial/ motor/ sensory**		3				3	
**Sensorimotor**		2					
**Temporal/ Amygdala/ iTEMP/FUS**			2	2			
**PCC/Thalamus**							
**Subcortical**							
**Insula**		anterior					
**Prefrontal**							

Supplementary Table 2: iCAP Studies used to compare human spatial iCAPs to monkey spatial iCAPs. For each study, green color indicates if iCAP was present and number indicates how many of that cluster was present.

iCAP	Lobe	Percent	Region	Z-Score	Voxels
**1**	Occipital_Lobe (Occipital)	98.8	middle_temporal_area (MT)	2	84
		82.5	preoccipital_visual_areas_2-3 (V2-V3)	1.99	1807
		87.1	visual_area_4 (V4)	1.99	671
		52.1	fundus_of_the_superior_temporal_sulcus (STSf)	1.96	232
		49.6	primary_visual_cortex (V1)	1.96	952
		51.7	ventromedial_intraparietal_sulcus (vm_IPS)	1.94	196
		96.2	visual_areas_V6_and_V6A (V6/V6A)	1.92	128
		95.9	area_TEO (TEO)	1.87	307
		20.7	hippocampal_formation (HF)	1.85	112

		28.9	area_7_(PGm)_on_the_medial_wall (area_7m)	1.81	65
		41.8	pulvinar_thalamus (Pul)	1.77	81
		86.3	medial_superior_temporal_area (MST)	1.76	126
**iCAP**	**Lobe**	**Percent**	**Region**	**Z-Score**	**Voxels**
**2**	telencephalon (tel)	100	secondary_somatosensory cortex (SII)	2.67	322
		97	floor_of_the_lateral_sulcus (floor_of_ls)	2.6	262
		83.3	claustrum (Cl)	2.36	250
		53	dorsal_striatum (DStr)	2.24	1126
		80.6	caudal_orbital_frontal_cortex (caudal_OFC)	2.2	325
		26.3	lateral_motor_cortex (M1/PM)	2.09	593
		96.7	primary_auditory_cortex (AI)	2.06	89
		30.3	primary_somatosensory_cortex (SI)	2.01	282
		82.4	core_areas_of_auditory_cortex (core)	1.96	145
		24.2	area_7_in_the_inferior_parietal_lobule (area_7_in_IPL)	1.89	157
		68.9	belt_areas_of_auditory_cortex (belt)	1.88	219
		27.2	caudal_superior_temporal_gyrus (STGc)	1.62	88
		36.2	septum_diagonal_band_complex (SDBR)	1.61	42
		23.9	rostral_superior_temporal_region (STGr/STSd)	1.61	164
		100	reticular_thalamus (Rt)	1.57	1
**iCAP**	**Lobe**	**Percent**	**Region**	**Z-Score**	**Voxels**

**3**	Frontal_Lobe (Frontal) Parietal_Lobe (Parietal)	72.5	primary_somatosensory_cortex (SI)	2.63	676
		100	midcingulate_cortex (MCC)	2.47	207
		73.5	lateral_motor_cortex (M1/PM)	2.42	1655
		81.3	area_5 (area_5)	2.29	335
		90.9	medial_supplementary_motor_areas (SMA/preSMA)	2.11	319
		52.6	lateral_intraparietal_sulcus (lat_IPS)	2	205
		32.4	area_7_(PGm)_on_the_medial_wall (area_7m)	1.98	73
		52.3	posterior_cingulate_gyrus (PCgG)	1.97	320
		24.2	anterior_cingulate_cortex (ACC)	1.93	126
		38.9	area_7_in_the_inferior_parietal_lobule (area_7_in_IPL)	1.82	252
		65.7	periarcuate_area_8A_(Frontal_Eye_Fields) (area_8A)	1.76	44
**iCAP**	**Lobe**	**Percent**	**Region**	**Z-Score**	**Voxels**
**4**	Frontal_Lobe (Frontal) telencephalon (tel)	100	medial_orbital_frontal_cortex (med_OFC)	2.37	294
		98.7	anterior_cingulate_cortex (ACC)	2.35	514
		99.4	lateral_orbital_frontal_cortex (lat_OFC)	2.31	702
		94	ventrolateral_prefrontal_cortex (vlPFC)	2.02	671
		53.5	dorsal_striatum (DStr)	1.93	1137
		61	medial_supplementary_motor_areas (SMA/preSMA)	1.89	214
		79.5	dorsolateral_prefrontal_cortex (dlPFC)	1.88	843
		35.2	ventral_striatum (VStr)	1.8	45

		59.3	caudal_orbital_frontal_cortex (caudal_OFC)	1.72	239
		23.7	midcingulate_cortex (MCC)	1.67	49
**iCAP**	**Lobe**	**Percent**	**Region**	**Z-Score**	**Voxels**
**5**	Temporal_Lobe (Temporal) telencephalon (tel)	99.2	rhinal_cortex (Rh)	2.91	386
		100	lateropallial_amygdala (lpAmy)	2.82	118
		96.4	temporal_pole (TG)	2.68	406
		100	ventropallial_amygdala (vpAmy)	2.57	42
		65	piriform_cortex (Pir)	2.34	26
		95.8	area_TE (TE)	2.33	1059
		100	endopiriform_claustrum (En)	2.3	32
		78.2	hippocampal_formation (HF)	2.27	423
		61.9	rostral_superior_temporal_region (STGr/STSd)	2.08	425
		41.6	fundus_of_the_superior_temporal_sulcus (STSf)	2.07	185
		30	claustrum (Cl)	2.05	90
		91.5	parahippocampal_cortex (paraHipp)	2.05	129
		63.6	medial_amygdala (mAmy)	1.89	35
		29.5	floculus_parafloculus (Fl-PFl)	1.62	52
**iCAP**	**Lobe**	**Percent**	**Region**	**Z-Score**	**Voxels**
**6**	myelencephalon (myel) metencephalon (met) mesencephalon (mes)	100	deep_cerebellar_nuclei (DCb)	3.1	40
		100	parabrachial_complex (PBC)	2.44	80
		100	intermediate_cerebellar_cortex (ICbCx)	2.43	1178
		96.8	vermis_cerebellar_cortex (VCbCx)	2.24	1331
		99.6	pontine_nucleus_region (Pn+)	2.21	268
		93.8	floculus_parafloculus (Fl-PFl)	2.16	165
		100	midbrain_tegmentum (TgMid)	2.14	47

		100	pontine_reticulum (retPons)	2.09	71
		91.1	lateral_cerebellar_cortex (LCbCx)	2.04	350
		57.5	colliculi (Co)	1.91	69
		89.3	periaqueductal_gray_region (PAGR)	1.85	75
		61.3	midbrain_reticulum (RtMid)	1.78	49
		22.4	preoccipital_visual_areas_2-3 (V2-V3)	1.76	491
		55.2	midbrain_dopaminergic_complex (DA_Mid)	1.74	133
**iCAP**	**Lobe**	**Percent**	**Region**	**Z-Score**	**Voxels**
**7**	Parietal_Lobe (Parietal)	100	area_7_(PGm)_on_the_medial_wall (area_7m)	3.17	225
		94.6	lateral_intraparietal_sulcus (lat_IPS)	2.86	369
		89.6	area_5 (area_5)	2.86	369
		74.8	area_7_in_the_inferior_parietal_lobule (area_7_in_IPL)	2.83	485
		97.1	ventromedial_intraparietal_sulcus (vm_IPS)	2.82	368
		33.9	visual_area_4 (V4)	2.6	261
		68.4	visual_areas_V6_and_V6A (V6/V6A)	2.45	91
		92.5	medial_superior_temporal_area (MST)	2.29	135
		65.9	middle_temporal_area (MT)	2.19	56
		24.8	caudal_superior_temporal_gyrus (STGc)	2.17	80
		72.4	posterior_cingulate_gyrus (PCgG)	2.16	443
		20.9	preoccipital_visual_areas_2-3 (V2-V3)	2.02	458
**iCAP**	**Lobe**	**Percent**	**Region**	**Z-Score**	**Voxels**

**8**	Frontal_Lobe (Frontal)	97.4	dorsolateral_prefrontal_cortex (dlPFC)	3.84	1033
		36.1	medial_orbital_frontal_cortex (med_OFC)	2.59	106
		29	lateral_motor_cortex (M1/PM)	2.55	653
		71.8	medial_supplementary_motor_areas (SMA/preSMA)	2.42	252
		66.8	ventrolateral_prefrontal_cortex (vlPFC)	2.41	477
		81.2	anterior_cingulate_cortex (ACC)	2.16	423
**iCAP**	**Lobe**	**Percent**	**Region**	**Z-Score**	**Voxels**
**9**	Occipital_Lobe (Occipital)	61.1	primary_visual_cortex (V1)	3.51	1171
		25.2	preoccipital_visual_areas_2-3 (V2-V3)	2.48	553
		24.9	vermis_cerebellar_cortex (VCbCx)	2.04	343
**iCAP**	**Lobe**	**Percent**	**Region**	**Z-Score**	**Voxels**
**10**	Occipital_Lobe (Occipital)	47	primary_visual_cortex (V1)	3.9	901
		20.3	preoccipital_visual_areas_2-3 (V2-V3)	2.8	444
		70.7	visual_areas_V6_and_V6A (V6/V6A)	2.05	94
**iCAP**	**Lobe**	**Percent**	**Region**	**Z-Score**	**Voxels**
**11**	metencephalon (met)	42.9	vermis_cerebellar_cortex (VCbCx)	4.56	590
		27.4	intermediate_cerebellar_cortex (ICbCx)	4.06	323
		37.8	lateral_cerebellar_cortex (LCbCx)	4.06	145

Supplementary Table 3: We compute the average percent coverage, z-score, and the total number of voxels occupied in brain areas defined with the CHARM and SARM atlas[1-3].

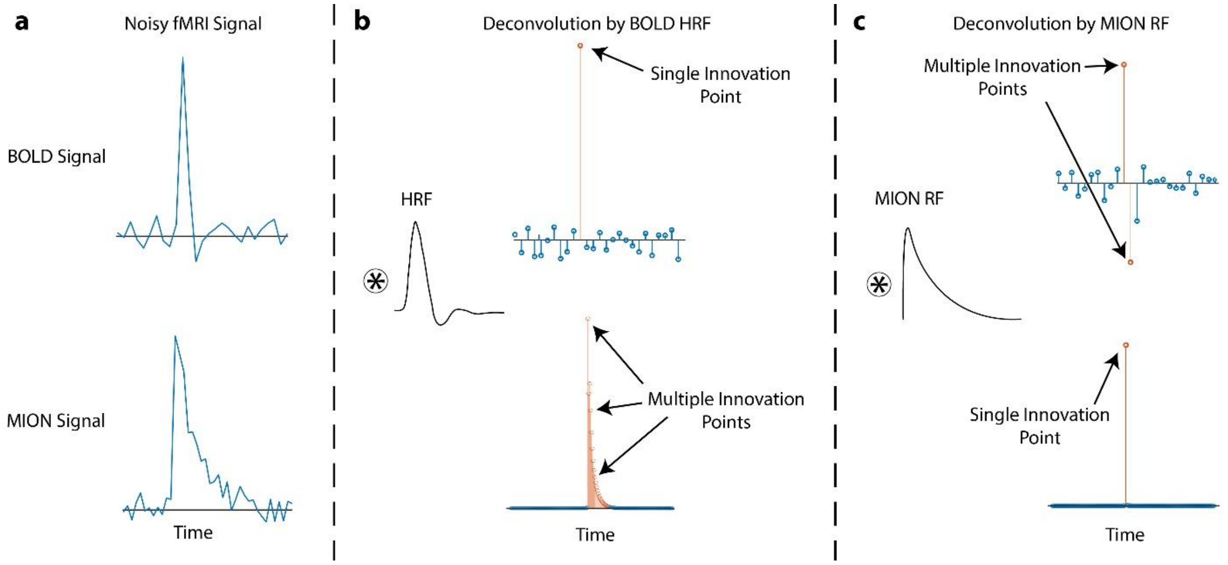

Supplementary Figure 1: BOLD HRF vs MION RF. A) Left column is representative of a noisy fMRI signal measured using BOLD and MION, respectively top and bottom. **B)** Middle column is the noisy fMRI signals deconvolved using the canonical BOLD HRF. **C)** Right column is the noisy fMRI signals deconvolved using the MION RF. It can be seen that the MION RF gets a much cleaner deconvolution of the MION signal (i.e., a single time innovation point) as compared to the BOLD HRF. Instead, the BOLD HRF gets a much cleaner deconvolution of the BOLD signal (i.e. a single time innovation point) as compared to the MION RF.

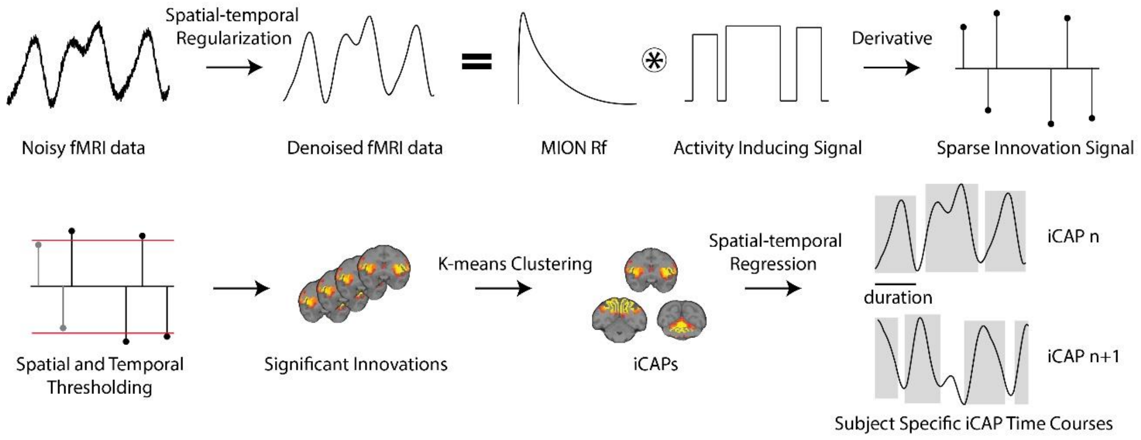

Supplementary Figure 2: Methodological pipeline of the iCAP framework. A) Noisy fMRI timecourses are denoised using a combined spatio-temporal regression, followed by a deconvolution from the MION rf to obtain a block type activity inducing signal, which is then differentiated to get the sparse innovation signal. The resulting innovation signals undergo a two-step spatial and temporal thresholding to select significant innovation frames. Then, the latter undergo a k-means clustering to obtain stable large-scale networks (i.e. iCAPs). The iCAPs are then back projected to the individual activity inducing signals using spatial-temporal regression to recover temporal profiles of each iCAP.

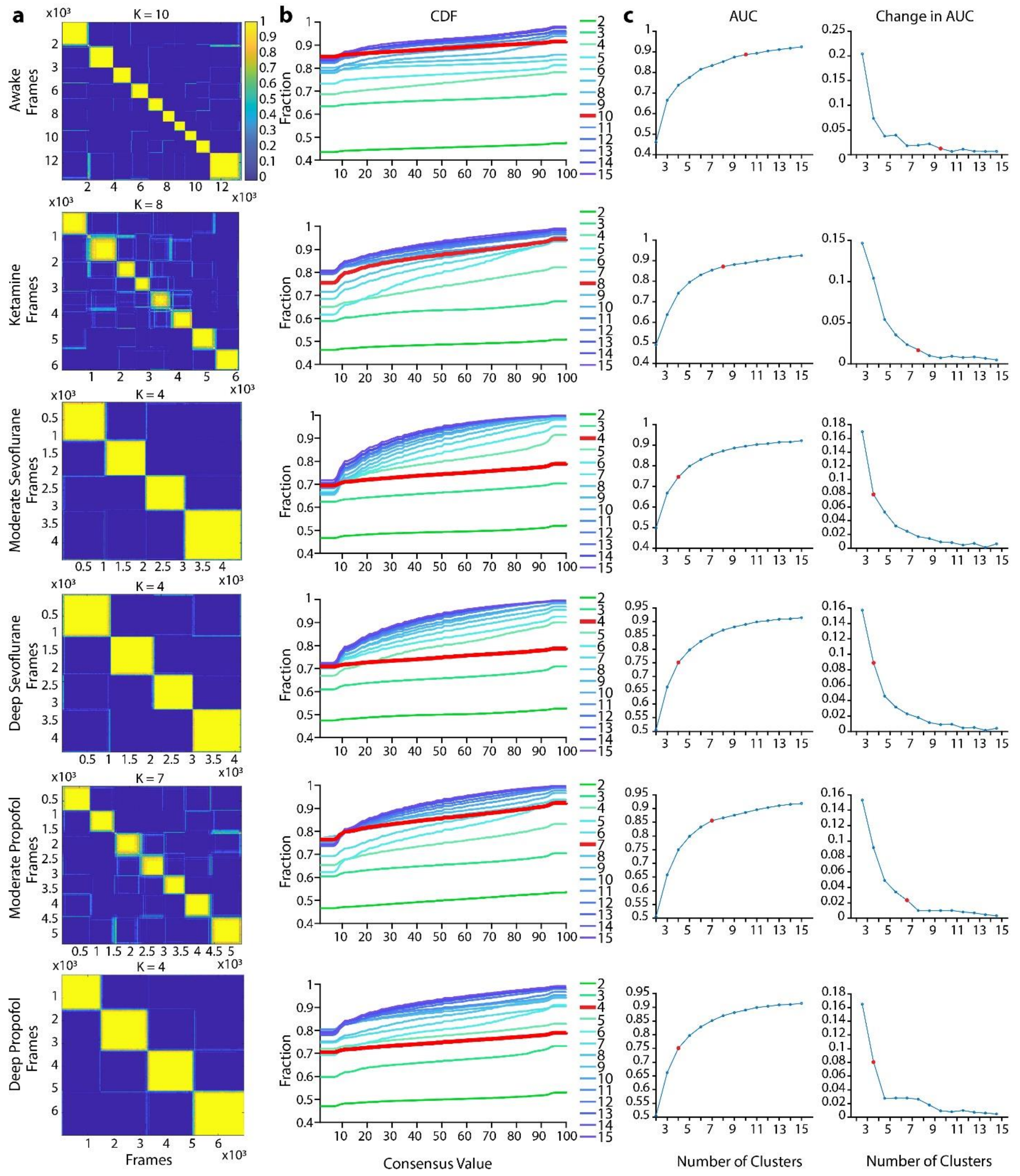

Supplementary Figure 3: Optimal Clustering for Each Condition Separately. A) Consensus clustering matrix for each condition; Awake, Ketamine, Moderate Sevoflurane, Deep Sevoflurane, Moderate Propofol, Deep Propofol. The x and y axes correspond to frame number. Values in the matrix range from 0 to 1, which indicate the reproducibility of the sampling across multiple runs, with 1 being perfectly re-sampled at all times. Diagonal values are expected to be equal to 1 (the same frame indices will always be clustered into the same group). **B)** The cumulative distribution function (CDF)

indicates the extent to which the consensus matrix distribution is skewed toward 0 and 1, with a flat line being the ideal shape (i.e. 0 means two frames are never clustered together while 1 means frames are always clustered together). In red CDF for n = 11 clusters. **C)** The area under the curve (AUC) of the CDF and the change in AUC display the optimal number of cluster K to which there is minimal increase in the AUC. In red the values chosen for each condition.

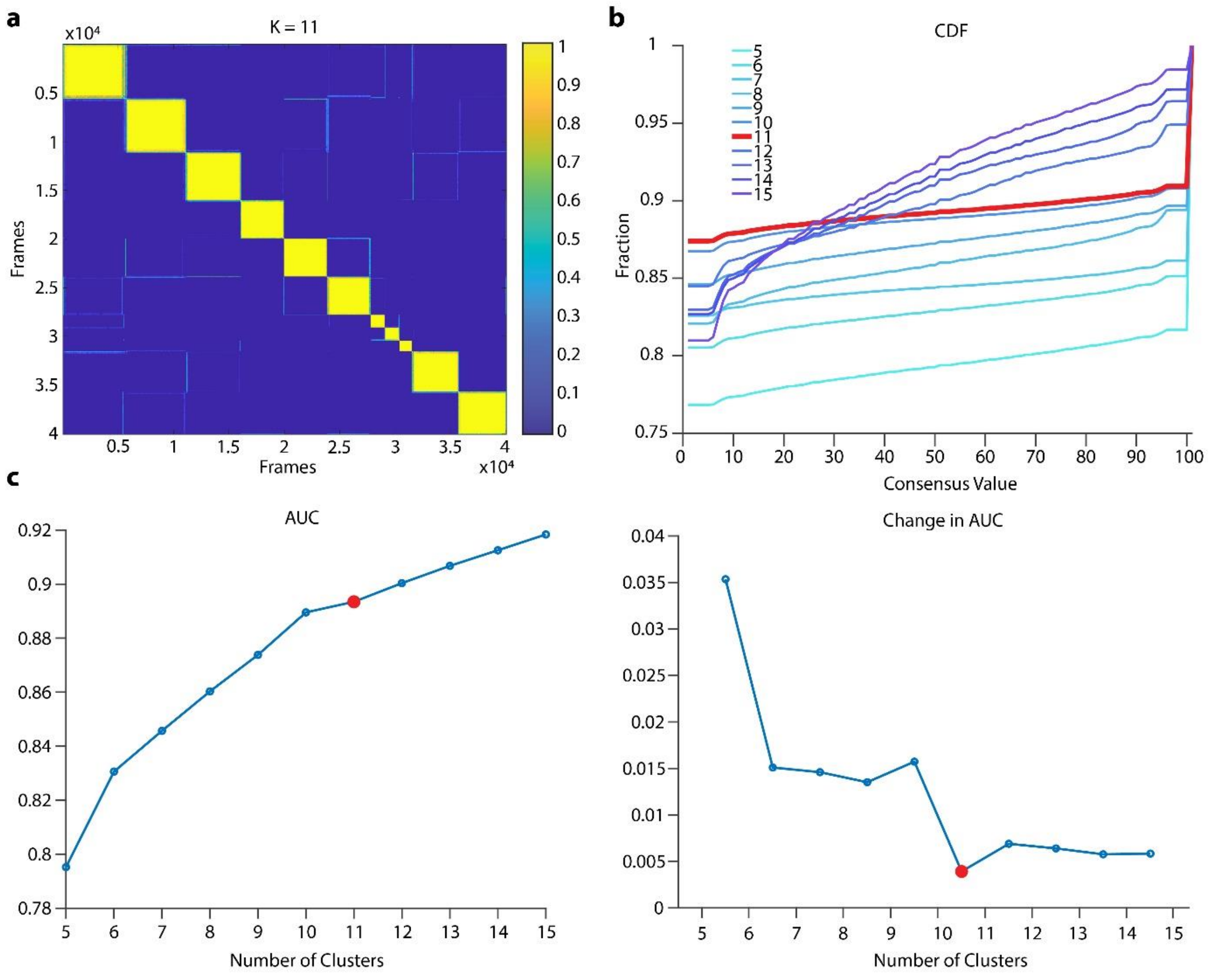

Supplementary Figure 4: Optimal Clustering. A) Consensus clustering matrix for n = 11 clusters. The x and y axes correspond to frame number. Values in the matrix range from 0 to 1, which indicate the reproducibility of the sampling across multiple runs, with 1 being perfectly re-sampled at all times. Diagonal values are expected to be equal to 1 (the same frame indices will always be clustered into the same group). **B)** The cumulative distribution function (CDF) indicates the extent to which the consensus matrix distribution is skewed toward 0 and 1, with a flat line being the ideal shape (i.e. 0 means two frames are never clustered together while 1 means frames are always clustered together). In red CDF for n = 11 clusters. **C)** The area under the curve (AUC) of the CDF and the change in AUC display the optimal number of cluster K to which there is minimal increase in the AUC. In red the values for n = 11 clusters.

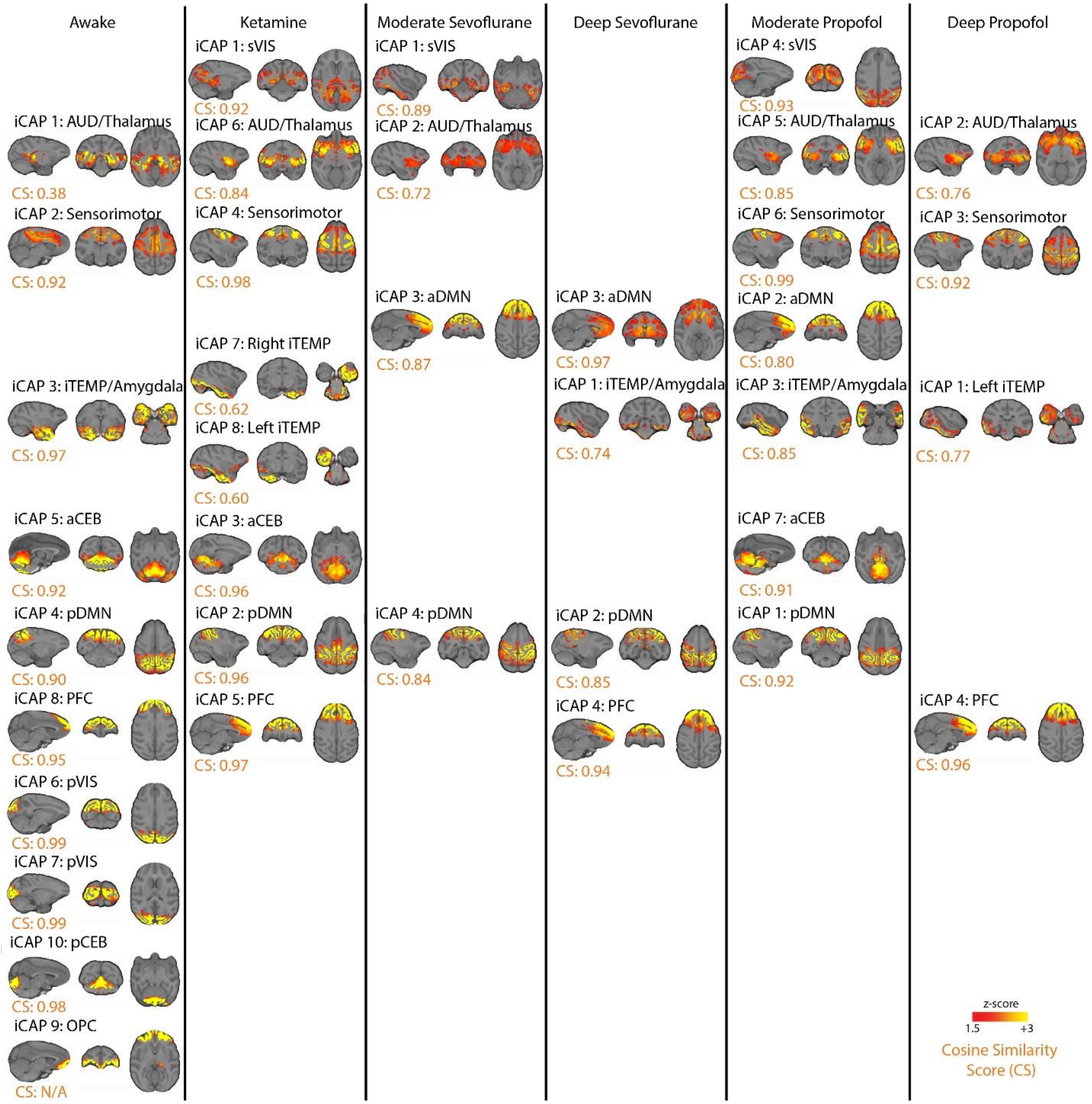

Supplementary Figure 5: Spatial iCAPs for Each Condition. Spatial pattern of the optimal iCAPs found when clustering significant innovation frames for each condition separately. The cosine similarity score for each iCAP compared to its counterpart in the all clustering condition is shown below each spatial map in orange font.

Supplemental References

1. Benjamin, J., et al., *A comprehensive macaque fMRI pipeline and hierarchical atlas.* bioRxiv, 2020: p. 2020.08.05.237818.
2. Hartig, R., et al., *The Subcortical Atlas of the Rhesus Macaque (SARM) for neuroimaging.* Neuroimage, 2021. **235**: p. 117996.
3. Reveley, C., et al., *Three-Dimensional Digital Template Atlas of the Macaque Brain.* Cereb Cortex, 2017. **27**(9): p. 4463-4477.
